# Supplementary material for: A computer vision based optical method for measuring fluid level in cell culture plates
Source: PLoS One. 2023 Sep 8;18(9):e0290951. doi: 10.1371/journal.pone.0290951 (PMC10490923; doi:10.1371/journal.pone.0290951)
Supplement: S1 Appendix — Detailed derivation of image distance transfer function. (PDF) [file pone.0290951.s001.pdf]

Appendix I

1. DERIVATION OF IMAGE DISTANCE FUNCTION

Labeled values are in reference to items in figure 1 of the main document

$$h = h_0 + L + h_1$$

since h_0 varies with L but h is constant we reframe h_0 as

$$h_0 = (h - h_1) - L$$

$h - h_1$ is constant and we will now label it as C_0

$$\begin{aligned}
x_0 &= (C_0 - L)\tan\theta_1 \\
C_1 &= \tan\theta_1 \\
x_1 &= x_0 + L\tan\theta_2 \\
C_2 &= \tan\theta_2 \\
x_f &= x_1 + h_1 C_1 \\
x_f &= (C_0 - L)C_1 + LC_2 + h_1 C_1 \\
x_f &= C_0 C_1 - LC_1 + LC_2 + h_1 C_1 \\
x_f &= L(-C_1 + C_2) + h_1 C_1 + C_0 C_1 \\
\tan(\theta_1) &= \frac{x_f}{I} \\
I &= \frac{x_f}{C_0} \\
I &= \frac{L(-C_1 + C_2) + h_1 C_1 + C_0 C_1}{C_0}
\end{aligned}$$
